# Effect of the Children’s Health Activity Motor Program on Motor Skills and Self-Regulation in Head Start Preschoolers: An Efficacy Trial

**DOI:** 10.3389/fpubh.2016.00173

**Published:** 2016-09-08

**Authors:** Leah E. Robinson, Kara K. Palmer, Kristen L. Bub

**Affiliations:** ^1^Child Movement Activity and Developmental Health Laboratory, School of Kinesiology, University of Michigan, Ann Arbor, MI, USA; ^2^Center for Human Growth and Development, University of Michigan, Ann Arbor, MI, USA; ^3^Department of Educational Psychology, College of Education, University of Illinois at Urbana-Champaign, Champaign, IL, USA

**Keywords:** intervention, motor skills, motor, school readiness, delay of gratification

## Abstract

Self-regulatory skills are broadly defined as the ability to manage emotions, focus attention, and inhibit some behaviors while activating others in accordance with social expectations and are an established indicator of academic success. Growing evidence links motor skills and physical activity to self-regulation. This study examined the efficacy of a motor skills intervention (i.e., the Children’s Health Activity Motor Program, CHAMP) that is theoretically grounded in Achievement Goal Theory on motor skill performance and self-regulation in Head Start preschoolers. A sample of 113 Head Start preschoolers (M_age_ = 51.91 ± 6.5 months; 49.5% males) were randomly assigned to a treatment (*n* = 68) or control (*n* = 45) program. CHAMP participants engaged in 15, 40-min sessions of a mastery climate intervention that focused on the development of motor skills over 5 weeks while control participants engaged in their normal outdoor recess period. The Delay of Gratification Snack Task was used to measure self-regulation and the Test of Gross Motor Development-2nd Edition was used to assess motor skills. All measures were assessed prior to and following the intervention. Linear mixed models were fit for both self-regulation and motor skills. Results revealed a significant time × treatment interaction (*p* < 0.001). In regard to motor skills, *post hoc* comparisons found that all children improved their motor skills (*p* < 0.05), but the CHAMP group improved significantly more than the control group (*p* < 0.001). Children in CHAMP maintained their self-regulation scores across time, while children in the control group scored significantly lower than the CHAMP group at the posttest (*p* < 0.05). CHAMP is a mastery climate movement program that enhance skills associated with healthy development in children (i.e., motor skills and self-regulation). This efficacy trial provided evidence that CHAMP helped maintain delay of gratification in preschool age children and significantly improved motor skills while participating in outdoor recess was not effective. CHAMP could help contribute to children’s learning-related skills and physical development and subsequently to their academic success.

## Introduction

Head Start is the largest federally sponsored early childhood education program in the United States and historically was developed to reduce socioeconomic disparities in school readiness (1). Currently, the percentage of children living in poverty has increased in the United States (2), and conditions associated with living in poverty (e.g., lack of desirable housing, family stress, and exposure to community violence) significantly contribute to poor school adjustment (3). Statistics also suggest that a growing percentage of American children, mainly those from families living in poverty, enter kindergarten lacking the skills (e.g., low self-regulatory skills) necessary for school success (4). These children are unprepared for the behavioral and learning demands of the classroom, and often experience poor academic outcomes that contribute to grade retention, early school dropout, and conflictual relationships with peers and teachers (5). Educational programs or interventions that target specific competencies, like self-regulatory skills, could positively influence school readiness outcomes in preschoolers (6). There is a need to support high-quality early childhood experiences or interventions that could contribute to school readiness, especially for children in low-income families (7).

Although self-regulation appears to be critical in predicting a range of outcomes, research in this area has been somewhat constrained by inconsistencies in how self-regulation has been defined. In general, self-regulation refers to the voluntary control of attentional, emotional, and behavioral impulses in accordance with a long-term goal (8, 9). Specifically, self-regulation measures a child’s ability to sustain his/her concentration and behavioral control while engaging in challenging tasks. More recently, self-regulation has been described as two distinct but related processes, including cognitive skills that facilitate working memory, response inhibition, planning, and attention shifting (i.e., executive functions) and behavioral skills that predispose individuals to more impulsive and immediately rewarding behaviors, including reactive under-control, sensation seeking, and delay of gratification (10). There is also some evidence to suggest that while young children possess all of these cognitive and behavioral skills, the skills act in a unified manner during early childhood and do not differentiate into distinct processes until later in childhood (11). Regardless of the exact definition used, self-regulation involves weighing a more appropriate response that typically aligns with an individual’s long-term goals against a more gratifying response that provides immediate satisfaction (12). Self-regulation has been shown to predict better school outcomes in preschool and elementary school (13), secondary school (14), and college (15). There is also a growing body of work linking self-regulation in childhood with health behaviors and health outcomes later in life (16–19). Evidence supports that children growing up in poverty and ethnic minority children, typically exhibit lower inhibitory control and delayed gratification along with increased problems associated with attention and working memory (20, 21). Thus, efforts to support the development of self-regulation skills for these children are particularly critical.

Self-regulatory skills develop rapidly between the ages of 2 and 5 years, as children enter preschool settings (22). Teachers consistently report that children are not entering kindergarten with the basic social–emotional skills needed to learn (4). As a result, efforts to design and test theoretically driven classroom- or curriculum-based programs that enhance these skills have increased dramatically in the past decade. The Chicago School Readiness Project (CSRP) targeted Head Start teachers’ classroom management behaviors and was effective in improving attention, impulse control, and executive function of preschoolers. Improvements were associated with better kindergarten readiness skills, including improved reading and mathematic skills as well as reduced behavior problems (6, 23). Programs that target self-regulation skills have also been found to lead to better health outcomes including weight loss maintenance (24) and healthier food choices (25) among youth.

Although there is evidence supporting the notion that self-regulation can be improved through interventions, very little research has examined this from a movement perspective. The body and brain work harmoniously together to understand and interpret the world around us, and the preschool years represent a period of rapid growth and development in both cognitive and motor skills. A recent systematic review found a weak-to-strong relationship between processes associated with self-regulation (i.e., cognitive skills) and motor skills in pediatric populations (26). Diamond (27) also concluded that preschoolers’ motor and cognitive skills are related in early learning and development. For instance, Becker et al. (28) found that young children’s fine motor skills were related to executive function (e.g., inhibitory control and working memory) and behavioral self-regulation (e.g., Head–Toes–Knees–Shoulders task). This finding supports a connection between motor skills and self-regulation that provides a strong rationale for using movement-based interventions to positively change self-regulation.

Lakes and Hoyt (29) found that compared to traditional Physical Education, a Tae Kwon Do approach lead to better self-regulation outcomes (working memory and inhibitory control) in elementary students. Palmer and colleagues (30) found that compared to a 30-min sedentary activity, an acute 30-min movement and physical activity-based intervention resulted in preschoolers demonstrating better sustained attention. These findings are promising and support that at least some aspects of self-regulation are malleable and can be enhanced through movement-based interventions.

This efficacy trial investigated the effect of an mastery climate motor skill intervention, the Children’s Health Activity Motor Program (CHAMP), on motor skill performance and self-regulation in Head Start preschoolers. We had two research questions: (a) does participation in CHAMP lead to greater gains in preschoolers’ motor skills? and (b) does participation in CHAMP lead to improvements or maintenance in preschoolers’ self-regulation? Based on research documenting the effectiveness of mastery climate motor skill interventions in children (31–33), we hypothesized that children in CHAMP would demonstrate significantly greater gains in motor skills over preschoolers in the control group. In regard to the second research question, we expected that children in CHAMP would exhibit improvements or maintenance in self-regulation over preschoolers in the control group. This hypothesis is based on research documenting the effectiveness of broader interventions that target self-regulation in children (34–36).

## Materials and Methods

### Participants and Settings

All participants were from a single Head Start center in the southeastern United States (*N* = 113, 45 girls, M_age_ = 52.4 ± 5.2 months; 80.5% African-American, 8.8% Caucasian American, 7.2% Hispanic, and 3.5% other). Children were divided into two groups: control/no treatment (*n* = 45, 18 girls, M_age_ = 51.6 ± 5.2 months) or an intervention group (*n* = 68, 27 girls, M_age_ = 52.4 ± 5.2 months). All children completed the motor skills assessment, and only a subsample completed the delay of gratification task (*n* = 65, 26 girls, M_age_ = 52.4 ± 5.3 months; 20 control, 45 treatment).

### Motor Skills

Motor skills were assessed with the Test of Gross Motor Development-2nd Edition [TGMD-2; (37)]. The TGMD-2 is a criterion- and norm-referenced standardized assessment used to measure fundamental motor skills in children aged 3–10 years old. The TGMD-2 assesses two broad categories of motor skills: locomotor skills – ability to propel the body through space and object control skills – ability to propel or manipulate objects with the hands and feet. The six locomotor skills assessed are run, jump, leap, hop, gallop, and slide; the six object control skills are throw, strike off a tee, catch, kick, roll, and dribble. For each skill, three to five performance skill criteria are measured. For example, one performance criterion for running was that “arms move in opposition to legs, elbows bent.” A “1” is awarded if the performance criteria is successfully completed, and a “0” is awarded it the performance criteria is not successfully completed. The highest total raw score a child can receive is a 96 (i.e., a maximum of 48 for both the locomotor and object control skill components). A higher score represents higher motor skill performance, whereas lower score indicates the absence of critical elements (i.e., lower motor skill performance). When testing, children are given a visual demonstration of a skill execution that includes all skill criteria followed by one practice trial and two test trials for each skill. All TGMD-2 test trials were video recorded and later coded by a single coder blind to the study. The coder had previously established inter-rater reliability of 97%. Mean test–retest reliability coefficients for the TGMD-2 subscales are 0.96 (locomotor) and 0.97 (object control).

### Self-Regulation

The delay of gratification snack task of the Preschool Self-Regulation Assessment was used to measure self-regulation (38). Delay of gratification is resisting a smaller more immediate reward in order to receive a larger reward later and is related to patience, impulse control, self-control, and willpower. For the delay of gratification snack task, children are instructed to keep their hands flat on the table. The researcher places a single snack object (i.e., goldfish cracker) under a clear cup in front of the child. The child is instructed to wait for the researcher’s cue before picking up the snack and placing it in another cup to save for later. This task is repeated four times using different delay periods – 10, 20, 30, and 15 s. Each time the child is given a numerical score based on their behaviors during the delay period: eats treat (1), touches treat (2), touches cup/timer (3), and waits for researcher’s cue before touching the snack (4). Snack task score ranges from 1 to 4, with higher score denoting better delaying capacity. The average scale score across all four delay periods was used in the final analyses.

### Children’s Health Activity Motor Program

CHAMP is a mastery focused, evidence-based intervention that enhances motor skills (31–33), perceived physical competence (33, 39), and physical activity (40). CHAMP is grounded in Achievement Goal Theory (41–45) and adheres to the TARGET structures [*t*ask, *a*uthority, *r*ecognition, *g*rouping, *e*valuation, and *t*ime; (41, 46), Table 1]. Achievement Goal Theory refers to our beliefs, attributions, and affect that contribute to our behaviors and represents the way individuals approach, engage, and respond to educational- and classroom-based activities (41, 43). Achievement goals are either *mastery-(task-)* or *performance-(ego-)oriented* (41, 44). Mastery learners are driven to learn and develop new skills, try to understand their work, improve their level of competence, and achieve a sense of mastery based on self-referenced standards. Performance learners focus their abilities and sense of self-worth on doing better than others, public recognition, surpassing normative-based standards, and achieving success with little effort.

**Table 1:** **Description of TARGET structures and CHAMP application**.

TARGET description	CHAMP alignment to TARGET structures	CHAMP link to self-regulation
Task: focuses on the presentation of the learning activities and tasks	A “slanted rope effect” is used to provide variety of tasks that range in level of difficulty to meet the skill level and ability of the learner along with their needs and interests of the learner	Learners self-select from a range of movement task and activities that vary in difficulty (low, moderate, and hard) (*SR skills* = *create goals and strategies, implement actions, plan actions and make decisions, self-manage behavior, self-monitor behavior, self-correct behavior*)
Authority: focuses on the interaction of the children and teacher within the learning environment with special consideration in classroom decision making	Authority or the “decision-making process” is fostered by allowing children to actively participate in choices and decisions that relate to learning	Learners are intrinsically driven to actively engage in environments that give them the opportunity to make decisions Learners have to self-manage and continually self-monitor their behaviors (*SR skills* = *create goals and strategies, plan actions and make decisions, self-monitor behavior, self-correct behaviors, manage emotions, understand and appropriately navigate social environments*)
Recognition: focuses on informal and formal rewards, incentives, and praise that are used and distributed by teachers to facilitate motivation	Avoid social comparison Recognize individual progress and improvement Recognition is private, the child’s sense of pride and satisfaction is derived from doing his/her best and not from outperforming others	Learners are encouraged to self-evaluate their own performance (*SR skills* = *self-monitor behaviors, self-reflection of progress*)
Grouping: focuses on grouping patterns	Children are not grouped, but will be given the opportunity to move freely and independently within the environment Allow the formation of heterogeneous cooperative groups that foster peer interaction (i.e., groups form and break up based on the individual desires of the child)	Learners self-select the people they engage with giving them the ability to self-govern their learning experience (*SR skills* = *plan actions and make decisions, self-monitor behavior, self-correct behaviors, manage emotions, understand and appropriately navigate social environments, collaborative efforts*)
Evaluation: focuses on methods that are used to assess, monitor, judge, and measure children’s behavior and learning	Evaluation and feedback are based on individual progress and improvement along with the process of learning movement rather than the product Involve children in self-evaluation Make evaluation private and meaningful	Learners are encourage to self-evaluate their own performance (*SR skills* = *working memory, self-monitor behaviors, self-reflection of progress, manage emotions, inhibition)*
Time: focuses on the workload, pace of instruction, and time allotment for children to complete learning activities and assignments	Teacher facilitates a learning experience that is tailored to the needs for the child Individualize instruction No set time allocated (e.g., schedule flexibility and vary pace of learning)	Child is allowed to self-direct their own learning (*SR skills* = *plan actions and make decisions, self-monitor behavior, self-correct behaviors, manage emotions*)

In a learning environment, achievement goals create an instructional climate (environment) that results in cognitive processes having “cognitive, affective, and behavioral consequences” [Ref. (47), p. 11]. Mastery-goal classrooms are associated with positive educational and achievement outcomes, like more effort contributes to success (48, 49); intrinsic interest and time on learning activities (50–52); positive attitudes toward learning (48, 51); and persistence in the face of difficulty (47). Mastery climates contribute to active engagement in the classroom that is characterized by the application of effective learning and problem solving strategies that could potentially enhance self-regulation. Self-regulation involves a child’s ability to self-monitor and self-correct their actions in behavior, motivation, and cognition (53). Thus, it is possible that self-regulation could be enhanced when children engage in mastery-oriented climates. These climates allow individuals to make their own decisions relating to learning tasks, to create goals and strategies, and to implement actions to meet goals within a learning context while managing their emotions, focusing their attention, and planning their actions.

CHAMP uses as mastery climate approach to provide children the opportunity to navigate a developmentally appropriate movement environment (32, 33, 41, 42). CHAMP is an evidence-based program that draws on effective instructional pedagogies from the physical education literature and principles that focus on critical elements and cue words of motor skills, effective modeling and demonstration, continuous and appropriate feedback, and repetitive cycling of motor skills and tasks. Newell’s constraints model is used to appropriately scaffold motor skills to promote motor skill acquisition (54). CHAMP targets children’s intrinsic motivation and persistence. Three theoretical tenets of Achievement Goal Theory are crucial to CHAMP: (a) the relationship between effort and personal progress, (b) learners’ self-selection of tasks, and (c) the environmental climate [Ref. (41, 42, 46); see Table 1 for a more detailed description of the alignment of these tenets with the TARGET structures, Ref. (55)].

CHAMP was implemented by two trained instructors in motor development – one Ph.D. researcher with 10 years of experience with implementing high-autonomy movement programs in pre- and elementary school settings and one Ph.D. student with 2 years of experience. Each session was 40 min in duration and consisted of (a) 2-min warm up designed to increase the heart and respiration rate, (b) 3–4 min of introductory activities where the motor skills were demonstrated, modeled, and the critical elements/cue words were instructed to the learners, (c) 20–25 min of motor skill engagement that adheres to the TARGET structures, (d) 5–7 min a large group activity that focused on reinforcing motor skills and increasing heart rate, and (e) 2–3 min of a closure activity that reinforced the critical elements and cue words of the motor tasks. For a more detailed description of the CHAMP intervention, refer to Ref. (31–33, 40).

### Procedures

Prior to the start of data collection, all experimental procedures were approved by the Office of Human Research Compliance Committee for Institutional Review Boards (IRBs) for the Protection of Human Subjects in Research – Social and Behavioral Research section. Parental consent was first obtained on all children, which was followed by child assent. Children were then randomly assigned to either a CHAMP treatment or control (outdoor/free-play recess) condition. Children in the CHAMP group replaced their outdoor recess with CHAMP 3 days/week for 5 weeks, and children in the control group did not make any changes in their daily routine. The control condition was the preschool’s typical movement program (i.e., outdoor/free-play recess) and was implemented according to the existing procedures within the preschool center. The center’s outdoor program consist of outdoor free-play activity on a large playground area with a variety of play structures (e.g., swings, slides, and ladders) that promote gross movement and activity in preschoolers. All movement sessions were 40 min in duration. The total dose of the CHAMP intervention was 15, 40-min sessions = 600 min. All children completed the delay gratification snack task and TGMD-2 prior to the start (pre) and after (post) the intervention.

### Statistical Analyses

Descriptive statistics (means and SDs) were obtained for delay of gratification and motor skills in both treatment groups. To mitigate potential statistical errors due to differences in sample sizes, main effects of time and treatment and potential time × treatment interactions were explored using linear mixed models. Separate linear mixed models were fit to determine changes in delay of gratification snack task and motor skills. Because of the autonomy supportive nature of the CHAMP intervention, the original model included only total TGMD-2 raw scores and secondary models were conducted for both locomotor and object control skill raw scores. Significant main effects and interactions were explored using *post hoc* paired or independent samples *t*-tests. All analyses were conducted in SPSS v.22. Alpha levels were set to 0.05 *a priori*.

## Results

### Motor Skills

Pre- and post-motor skills assessments were measured on 113 children (68 CHAMP and 45 control). See Table 2 for full descriptive statistics.

**Table 2:** **Full descriptive statistics for motor skills**.

	CHAMP (*n* = 68)			Control (*n* = 45)		
	Total	Locomotor	Object control	Total	Locomotor	Object control
Pre	17.69 (9.78)	8.71 (5.38)	8.98 (5.58)	17.00 (9.87)	8.20 (5.56)	8.68 (5.64)
Post	60.19 (19.69)	29.12 (10.07)	31.07 (10.27)	25.01 (16.48)	10.93 (6.62)	14.68 (10.32)
Difference	42.50 (15.54)	20.41 (7.95)	22.09 (8.85)	8.71 (16.10)	2.73 (7.10)	6.00 (9.81)

#### Main Effects

**Figure 1:**
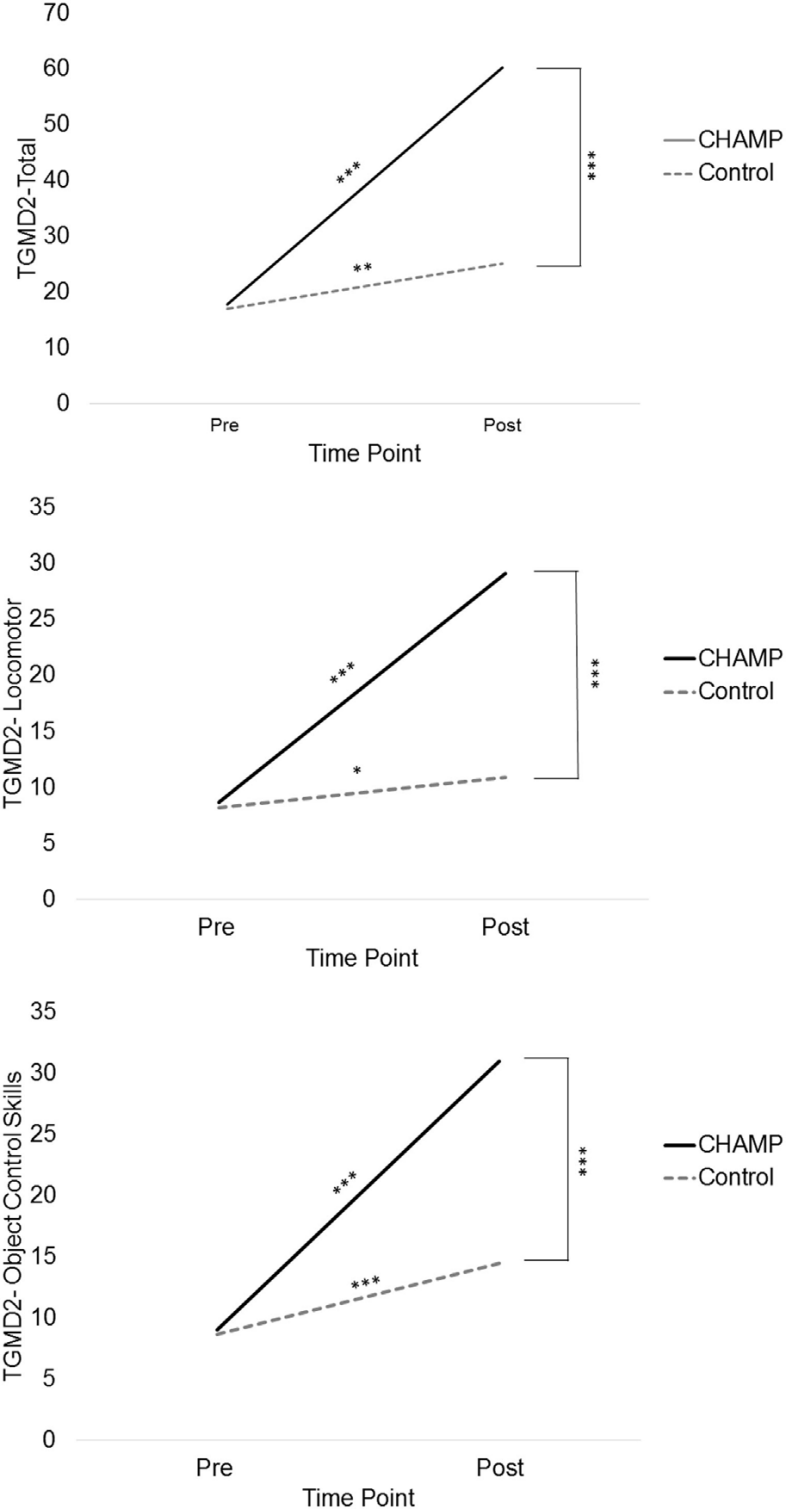
**Total, locomotor, and object control skill scores across time according to treatment group**.

The linear mixed model fit for total TGMD-2 scores revealed a significant main effect of time [*F*_(1,111)_ = 278.34, *p* < 0.001] and treatment [*F*_(1,111)_ = 55.45, *p* < 0.001] as well as a significant time × treatment interaction [*F*_(1,111)_ = 129.35, *p* < 0.001; see Figure 1]. The secondary analysis for locomotor and object control skills found similar main effects of time [*F*_(1,110.8)_ = 245.22, *p* < 0.001 and *F*_(1,111.17)_ = 249.14, *p* < 0.001, respectively], treatment [*F*_(1,111.3)_ = 58.52, *p* < 0.001 and *F*_(1,111.5)_ = 40.1, *p* < 0.001, respectively], and time × treatment interaction [*F*_(1,110.8)_ = 145.22, *p* < 0.001 and *F*_(1,111.17)_ = 81.58, *p* < 0.001, respectively; see Figure 1]. Independent *t*-tests were used to explore simple effects of treatment, and paired sample *t*-tests were used to explore simple effects of time.

#### Simple Effects

##### Treatment

There were no significant between group differences at pretest in regard to total TGMD-2 scores [17.00 ± 9.87 vs. 17.69 ± 9.78, *t*_(111)_ = −0.37, *p* = 0.72], locomotor scores [8.20 ± 5.56 vs. 8.71 ± 5.38, *t*_(111)_ = −0.36, *p* = 0.72], or object control scores [8.68 ± 5.64 vs. 8.98 ± 5.58, *t*_(111)_ = −0.30, *p* = 0.77]. At posttest, the treatment group had significantly higher total scores [25.01 ± 16.48 vs. 60.19 ± 19.69, *t*_(111)_ = 9.90, *p* < 0.001, *d* = 1.94], locomotor scores [10.93 ± 6.62 vs. 29.12 ± 10.07, *t*_(110)_ = 10.58, *p* < 0.001, *d* = 2.13], and object control scores [14.68 ± 10.32 vs. 31.07 ± 10.27, *t*_(111)_ = 8.23, *p* < 0.001, *d* = 1.59].

##### Time

The control group exhibited significant improvements from pre- to posttest in total TGMD-2 scores [17.00 ± 9.87 to 25.01 ± 16.48, *t*_(44)_ = 3.35, *p* < 0.01, *d* = 0.61], locomotor [8.20 ± 5.56 to 10.93 ± 6.62, *t*_(43)_ = 2.55, *p* < 0.05, *d* = 0.45], and object control skills [8.68 ± 5.64 to 14.68 ± 10.32, *t*_(43)_ = 4.06, *p* < 0.001, *d* = 0.75]. The CHAMP group also showed significant improvements from pre- to posttest in total TGMD-2 scores [17.69 ± 9.78 to 60.19 ± 19.69, *t*_(67)_ = 22.55, *p* < 0.001, *d* = 2.88], locomotor [8.71 ± 5.38 to 29.12 ± 10.07, *t*_(67)_ = 21.17, *p* < 0.001, *d* = 2.64], and object control skills scores [8.98 ± 5.58 to 31.07 ± 10.27, *t*_(67)_ = 20.59, *p* < 0.001, *d* = 2.79].

#### Total Change

**Figure 2:**
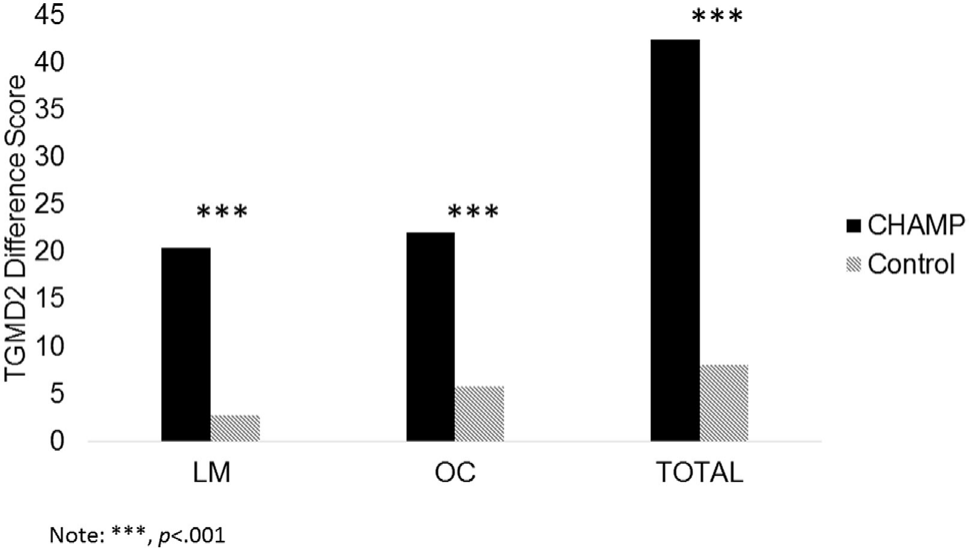
**Differences between groups in motor skills change scores**.

To determine total changes in motor skills across time, a change score was calculate by subtracting the pre- from the posttest for the total TGMD-2, locomotor, and object control scores (see Figure 2). Independent samples *t*-tests were conducted to determine if differences in motor skill changes were present between groups (i.e., CHAMP vs. control). Results showed that children in the control group did not improve as much as children in the CHAMP group in total TGMD-2 [8.71 ± 16.10 vs. 42.50 ± 15.54, *t*_(111)_ = −11.37, *p* < 0.001, *d* = 2.14], locomotor [2.73 ± 7.10 vs. 20.41 ± 7.95, *t*_(110)_ = −11.98, *p* < 0.001, *d* = 2.35], and object control scores [6.00 ± 9.81 to 22.09 ± 8.85, *t*_(110)_ = −9.01, *p* < 0.001, *d* = 1.72].

### Self-Regulation

A total of 45 (17 girls and 28 boys, M_age_ = 52.4 ± 5.3 months) children in CHAMP and 20 children in the control (9 girls and 11 boys, M_age_ = 52.5 ± 5.3 months) completed both the pre- and posttest delay of gratification task. See Table 3 for full descriptive statistics.

**Table 3:** **Full descriptive statistics for delay of gratification**.

	CHAMP (*n* = 45)	Control (*n* = 22)
Pre	3.80 (0.38)	3.57 (0.66)
Post	3.79 (0.49)	3.08 (1.29)

#### Main Effects

**Figure 3:**
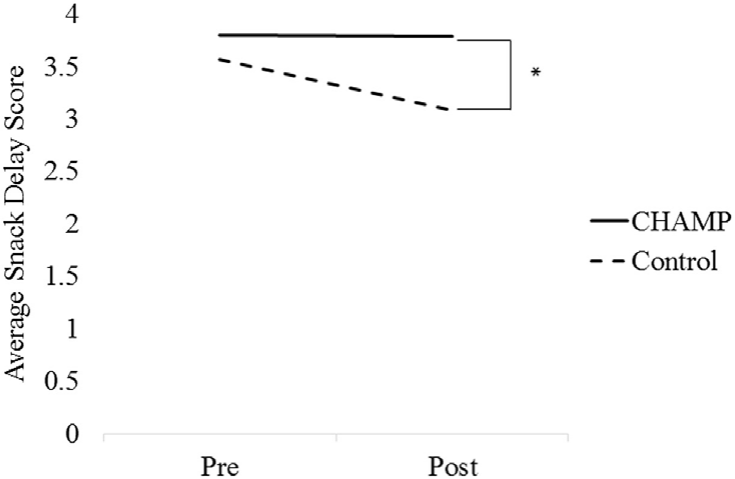
**Delay of gratification scores across time according to treatment group**.

The linear mixed model revealed a main effect of treatment where the control group demonstrated significantly lower delay of gratification scores compared to the treatment group [−0.72, *t*_(65.76)_ = −4.03, *p* < 0.001]. The model also found a significant treatment × time interaction [0.49, *t*_(65.76)_ = 2.31, *p* < 0.05; see Figure 3]. The model did not find a significant main effect of time.

#### Simple Effects

*Post hoc t*-tests revealed that at the pretest there were no significant differences between the treatment and control groups [3.80 ± 0.38 vs. 3.57 ± 0.66, *t*_(27.74)_ = −1.50, *p* > 0.05] but significant differences were present at posttest [3.79 ± 0.49 vs. 3.08 ± 1.29, *t*_(21.45)_ = −2.41, *p* < 0.05, *d* = 0.73].

## Discussion

The present study focused on the effects of a mastery climate movement program, CHAMP that has been shown to promote motor skills, perceived motor competence, and physical activity in young children (31–33, 39, 40). This intervention efficacy trial tested the treatment effects of preschoolers’ participation in CHAMP or outdoor recess/free-play (control) on motor skill performance and self-regulation skills. Significant treatment effects were found for both motor skills and self-regulation scores. Specifically, children in CHAMP demonstrated significantly greater motor skill performance and also maintained high self-regulation scores compared with the control.

### CHAMP and Motor Skills Outcomes

Motor skills are necessary for children to independently navigate their environment (32, 33, 56) and are the foundation for more complex movement (57, 58). Motor skills have an essential role in supporting positive developmental trajectories of health (58). Despite the fact that motor skills support healthy development, the inclusion and instruction of motor skills in early childhood programs are often non-existent. However, findings from previous work have shown that developmentally appropriate and well-designed movement programs are effective in promoting motor skills (31, 32, 59). Further evidence also supports that children who receive no formalized instruction demonstrate no improvements in these skills (31, 32, 59).

Our first hypothesis aligned with the previous literature (31, 32, 59). Specifically, we found that all children significantly improved their motor skills across time but the greatest gains were seen in children who completed CHAMP. CHAMP participants demonstrated a ~42 points improvement in TGMD-2 raw scores while control participants only exhibited an ~8 point improvement. This is a difference of 34 points. As a reminder, the children demonstrated similarly motor skills at pretest and differences were only present following the intervention – CHAMP participants exhibited motor skills at the 76th%tile, while the control group was at the 10th%tile. These findings support that motor skills are not naturally occurring behaviors but are skills that need to be taught, practiced, and reinforced with high-quality instruction and feedback.

### CHAMP and Self-Regulation Outcomes

Self-regulatory skills are also important and aid in healthy child development. These skills allow individuals to monitor and control their behavior, emotions, or thoughts and alter them based on the demands of the current situation. Research supports that these skills predict school success and achievement (60). With self-regulation occurring during the early childhood years, there is a need for effective intervention programs. The theoretical foundation and implementation structure of the CHAMP intervention aligns with both cognitive and behavioral components of self-regulation, making it a potential avenue to simultaneously enhance self-regulation and health outcomes in this population.

We predicted that the CHAMP participants would exhibit greater gains or maintenance in self-regulation over preschoolers in the control group. The children began the intervention with similar scores on the delay of gratification snack task, but our findings indicate that the control group scored significantly lower than CHAMP participants after the intervention. These findings provide great promise as it relates to the potential use of CHAMP to maintain self-regulation in preschoolers. In this current efficacy trial, it was difficult for the preschoolers’ delay of gratification scores to increase since they scored close to the maximum at pretest (i.e., ~3.8 out of 4.0). However, the CHAMP participants self-regulation (i.e., delaying capacity) was maintained across the 5-week period. The TARGET structures that were manipulated within CHAMP to create a mastery climate appear to support self-regulatory skills. For example, students were given authority and responsibility to decide how they engaged in the movement environment as it related to the task activities they chose, level of difficulty, time allotment (time management), and grouping (peer interaction). They also had to self-evaluate their own progress/performance and self-manage their behavior (Table 1 provides a detailed description of the TARGET structure and alignment with CHAMP and self-regulation). These behaviors align with the behavioral regulation (i.e., inhibition of an immediate, impulse control, and inappropriate behavior) skills that are necessary to complete the delay of gratification task.

There could be several reasons why we did not see an increase in self-regulation scores in the current study. Lakes and Hoyt (29) used a Tae Kwon Do treatment during physical education to promote self-regulation in K – 5th graders and found positive improvements in working memory and inhibitory control. The intervention dose was a total of 1080 min (26, 45 min sessions over 3 months) compared to the CHAMP treatment that was 600 min (15, 40-min sessions over 5 weeks). Other early childhood intervention studies that are not movement-based have also seen benefits from a treatment with a larger dose (61, 62). To the best of our knowledge, this is the first movement-based intervention that addressed this question in preschoolers. Future work examining the impact of movement-based interventions to promote self-regulation outcomes is needed. The CHAMP intervention appeared to be effective in maintaining self-regulation which was a positive finding, but the sample size could have also been another limiting factor.

### Limitations and Future Research

Although the present study supports the preliminary efficacy of a mastery-based movement program, CHAMP, on motor skill performance and self-regulation in preschool age children, there are some limitations. One limitation of this study was that only one measure, delay of gratification snack task, was used to assess self-regulation in the preschoolers. In an ideal experiment, a combination of direct measures, teacher reports, and classroom observations would be used to provide a comprehensive understanding of children’s self-regulation skills. Due to the fact that this was an efficacy trial, it was not feasible but future work should incorporate a board range of assessments. Additionally, various constraints within the preschool schedule (e.g., field trip, weather closures, and delays), we were unable to conduct pretest delay of gratification assessments on all of the children which contributed to the smaller sample size. This was beyond our control and a limitation of the efficacy trial.

One may argue that an outdoor recess (free-play) is not a true control. But, it is quite difficult to withhold the standard practice in an early childhood programs when the intervention is a movement program. There is a vast amount of evidence from the motor development literature that clearly establishes the use of outdoor recess (free-play) as control group for intervention studies due to the fact that children in these groups see no improvements in this motor skills (31, 32, 59), physical competence (33, 39), or physical activity participation (40). In this efficacy trial, the control participants (i.e., outdoor recess/free-play) demonstrated a significant decline in self-regulation when everything in their preschool day was held consistent to their CHAMP counterparts. Therefore, we assume that the mastery climate, CHAMP intervention was a determining factoring that positively affected the children’s delayed of gratification.

Future studies should also consider other aspects of the interventions that could also affect children’s self-regulation. For this efficacy trial, no data were assessed on the classroom and home environment. Information regarding the classroom teachers, classroom environment, parenting style, and home environment would have been useful, since these factors have a significance influence on the development of children’s self-regulation (63).

### Practical Implications and Conclusions

There is a growing priority to promote motor skill competence in children as it contributes to positive health trajectories (57, 58). CHAMP delivers a high-quality movement program in a mastery climate environment. CHAMP is an evidence-based intervention that enhances motor skills, perceived motor competence, and physical activity in children (31–33, 39, 40). This efficacy trial provides evidence that CHAMP also aids in maintaining a key competency that is associated with school readiness outcomes in preschoolers’ (i.e., delay of gratification). The present study has the potential to shape and inform preschool curricula as a means of integrating movement education and school readiness that will help preschoolers enter school healthy, activity, and ready to learn.

## Author Contributions

LR conceptualized and designed the study. LR and KP implemented the intervention and data acquisition. LR and KP completed data analyses and interpretation. LR, KP, and KB contributed to the writing of the manuscript. LR, KP, and KB agreed with manuscript results and conclusions. All authors reviewed, made critical revisions, and approved of the final manuscript.

## Conflict of Interest Statement

The authors declare that the research was conducted in the absence of any commercial or financial relationships that could be construed as a potential conflict of interest.

## References

[1] United States Department of Health and Human Services. Head Start Impact Study: First Year Findings. Washington, DC (2005).

[2] Douglas-Hall A Chau M Koball H Basic Facts about Low-Income Children: Birth to Age 18. New York: National Center for Children in Poverty (2008).

[3] McLoyd VC Socioeconomic disadvantage and child development. Am Psychol (1998) 53(2):185–204. 10.1037/0003-066X.53.2.185 9491747

[4] Rimm-Kaufman SE Pianta RC Cox MJ Teachers’ judgments of problems in the transition to kindergarten. Early Child Res Q (2000) 15(2):147–66. 10.1016/S0885-2006(00)00049-1

[5] Ryan R Fauth R Brooks-Gunn J Childhood poverty: implications for school readiness and early childhood education. In: Spodek B Saracho ON, editors. Handbook of Research on the Education of Children [Internet]. Mahwah, NJ: Erlbaum Associates (2006). p. 323–46.

[6] Raver CC Jones SM Li-Grining C Zhai F Bub K Pressler E CSRP’s impact on low-income preschoolers’ preacademic skills: self-regulation as a mediating mechanism. Child Dev (2011) 82(1):362–78. 10.1111/j.1467-8624.2010.01561.x 21291447 PMC3682645

[7] Shonkoff J Phillips D From Neurons to Neighborhoods. Washington, DC: National Academy Press (2000). 25077268

[8] Duckworth AL Carlson SM Self-regulation and school success. In: Sokol B Grouzet F Muller U, editors. Self-Regulation and Autonomy: Social and Developmental Dimensions of Human Conduct. Cambridge, England: Cambridge University Press (2013). p. 208–30.

[9] Raver CC. Placing emotional self-regulation in sociocultural and socioeconomic contexts. Child Dev (2004) 75(2):346–53. 10.1111/j.1467-8624.2004.00676.x 15056189

[10] Duckworth AL Steinberg L. Unpacking self-control. Child Dev Perspect (2015) 9(1):32–7. 10.1111/cdep.12107 25821515 PMC4372146

[11] Zelazo PD Carlson SM Hot and cool executive function in childhood and adolescence: development and plasticity. Child Dev Perspect (2012) 6(4):354–60.

[12] Duckworth AL Kern ML. A meta-analysis of the convergent validity of self-control measures. J Res Pers (2011) 45(3):259–68. 10.1016/j.jrp.2011.02.004 21643479 PMC3105910

[13] Evans GW Rosenbaum J Self-regulation and the income-achievement gap. Early Child Res Q (2008) 23(4):504–14. 10.1016/j.ecresq.2008.07.002

[14] Pagani LS Vitaro F Tremblay RE McDuff P Japel C Larose S When predictions fail: the case of unexpected pathways toward high school dropout. J Soc Issues (2008) 64(1):175–94. 10.1111/j.1540-4560.2008.00554.x

[15] McClelland MM Acock AC Piccinin A Rhea SA Stallings MC. Relations between preschool attention span-persistence and age 25 educational outcomes. Early Child Res Q (2013) 28(2):314–24. 10.1016/j.ecresq.2012.07.008 23543916 PMC3610761

[16] Appleton AA Buka SL McCormick MC Koenen KC Loucks EB Gilman SE Emotional functioning at age 7 years is associated with C-reactive protein in middle adulthood. Psychosom Med (2011) 73(4):295. 10.1097/PSY.0b013e31821534f6 21536835 PMC3090487

[17] Bub KL Robinson LE Curtis D Longitudinal associations between self-regulation and health across early and middle childhood. Health Psychol (2016). 10.1037/hea0000401 PMC5067975 27513478

[18] Schlam TR Wilson NL Shoda Y Mischel W Ayduk O. Preschoolers’ delay of gratification predicts their body mass 30 years later. J Pediatr (2013) 162(1):90–3. 10.1016/j.jpeds.2012.06.049 22906511 PMC3504645

[19] Tsukayama E Toomey SL Faith MS Duckworth AL. Self-control as a protective factor against overweight status in the transition from childhood to adolescence. Arch Pediatr Adolesc Med (2010) 164(7):631–5. 10.1001/archpediatrics.2010.97 20603463 PMC2914627

[20] Blair C. Stress and the development of self-regulation in context. Child Dev Perspect (2010) 4(3):181–8. 10.1111/j.1750-8606.2010.00145.x 21779305 PMC3138186

[21] Evans GW Gonnella C Marcynyszyn LA Gentile L Salpekar N. The role of chaos in poverty and children’s socioemotional adjustment. Psychol Sci (2005) 16(7):560–5. 10.1111/j.0956-7976.2005.01575.x 16008790

[22] McClelland MM Morrison FJ The emergence of learning-related social skills in preschool children. Early Child Res Q (2003) 18(2):206–24. 10.1016/S0885-2006(03)00026-7

[23] Jones SM Bub KL Raver CC. Unpacking the black box of the CSRP intervention: the mediating roles of teacher-child relationship quality and self-regulation. Early Educ Dev (2013) 24(7):1043. 10.1080/10409289.2013.825188 24729666 PMC3979484

[24] Verbeken S Braet C Goossens L Van der Oord S. Executive function training with game elements for obese children: a novel treatment to enhance self-regulatory abilities for weight-control. Behav Res Ther (2013) 51(6):290–9. 10.1016/j.brat.2013.02.006 23524063

[25] Riggs NR Sakuma K-LK Pentz MA. Preventing risk for obesity by promoting self-regulation and decision-making skills pilot results from the PATHWAYS to health program (PATHWAYS). Eval Rev (2007) 31(3):287–310. 10.1177/0193841X06297243 17478630

[26] van der Fels IM te Wierike SC Hartman E Elferink-Gemser MT Smith J Visscher C The relationship between motor skills and cognitive skills in 4-16 year old typically developing children: a systematic review. J Sci Med Sport (2015) 18(6):697–703. 10.1016/j.jsams.2014.09.007 25311901

[27] Diamond A. Close interrelation of motor development and cognitive development and of the cerebellum and prefrontal cortex. Child Dev (2000) 71(1):44–56. 10.1111/1467-8624.00117 10836557

[28] Becker DR McClelland MM Loprinzi P Trost SG Physical activity, self-regulation, and early academic achievement in preschool children. Early Educ Dev (2014) 25(1):56–70. 10.1080/10409289.2013.780505

[29] Lakes KD Hoyt WT Promoting self-regulation through school-based martial arts training. J Appl Dev Psychol (2004) 25(3):283–302. 10.1016/j.appdev.2004.04.002

[30] Palmer KK Miller MW Robinson LE Acute exercise enhances preschoolers’ ability to sustain attention. J Sport Exerc Psychol (2013) 35(4):433–7. 10.1123/jsep.35.4.433 23966452

[31] Veldman SL Palmer KK Okely AD Robinson LE Promoting ball skills in preschool-aged girls. J Sci Med Sport (2016). 10.1016/j.jsams.2016.04.009 27283343

[32] Robinson LE Goodway JD. Instructional climates in preschool children who are at-risk. Part I: object-control skill development. Res Q Exerc Sport (2009) 80(3):533–42. 10.5641/027013609X13088500159480 19791639

[33] Robinson LE Effect of a mastery climate motor program on object control skills and perceived physical competence in preschoolers. Res Q Exerc Sport (2011) 82(2):355–9. 10.1080/02701367.2011.10599764 21699116

[34] Diamond A Barnett WS Thomas J Munro S Preschool program improves cognitive control. Science (2007) 318(5855):1387–8. 10.1126/science.1151148 18048670 PMC2174918

[35] Domitrovich CE Cortes RC Greenberg MT Improving young children’s social and emotional competence: a randomized trial of the preschool “PATHS” curriculum. J Prim Prev (2007) 28(2):67–91. 10.1007/s10935-007-0081-0 17265130

[36] Tominey SL McClelland MM Red light, purple light: findings from a randomized trial using circle time games to improve behavioral self-regulation in preschool. Early Educ Dev (2011) 22(3):489–519. 10.1080/10409289.2011.574258

[37] Ulrich DA Test of Gross Motor Development-2. Austin: Prod-Ed (2000).

[38] Smith-Donald R Raver CC Hayes T Richardson B Preliminary construct and concurrent validity of the preschool self-regulation assessment (PSRA) for field-based research. Early Child Res Q (2007) 22(2):173–87. 10.1016/j.ecresq.2007.01.002

[39] Robinson LE Rudisill ME Goodway JD. Instructional climates in preschool children who are at-risk. Part II: perceived physical competence. Res Q Exerc Sport (2009) 80(3):543–51. 10.5641/027013609X13088500159480 19791640

[40] Palmer KK Matsuyama AL Robinson LE Impact of structured movement time on preschoolers’ physical activity engagement. Early Child Educ J (2016):1–6. 10.1007/s10643-016-0778-x

[41] Ames C Classrooms: goals, structures, and student motivation. J Educ Psychol (1992) 84(3):261 10.1037/0022-0663.84.3.261

[42] Ames C Achievement goals, motivational climate, and motivational processes. In: Roberts GC, editor. Motivation in Sport and Exercise. Champaign, IL: Human Kinetics (1995).

[43] Dweck CS Leggett EL A social-cognitive approach to motivation and personality. Clin Psychol Rev (1988) 95(2):256.

[44] Nicholls JG The Competitive Ethos and Democratic Education. Cambridge, MA: Harvard University Press (1989).

[45] Nicholls JG Achievement motivation: conceptions of ability, subjective experience, task choice, and performance. Clin Psychol Rev (1984) 91(3):328.

[46] Epstein JL Family structures and student motivation: a developmental perspective. In: Ames C Ames R, editors. Research on Motivation in Education. San Diego, CA: Academic Press (1989). p. 259–95.

[47] Elliott ES Dweck CS. Goals: an approach to motivation and achievement. J Pers Soc Psychol (1988) 54(1):5. 10.1037/0022-3514.54.1.5 3346808

[48] Ames C Archer J Achievement goals in the classroom: students’ learning strategies and motivation processes. J Educ Psychol (1988) 80(3):260 10.1037/0022-0663.80.3.260

[49] Nicholls JG Patashnick M Nolen SB Adolescents’ theories of education. J Educ Psychol (1985) 77(6):683 10.1037/0022-0663.77.6.683

[50] Butler R Task-involving and ego-involving properties of evaluation: effects of different feedback conditions on motivational perceptions, interest, and performance. J Educ Psychol (1987) 79(4):474 10.1037/0022-0663.79.4.474

[51] Meece JL Blumenfeld PC Hoyle RH Students’ goal orientations and cognitive engagement in classroom activities. J Educ Psychol (1988) 80(4):514 10.1037/0022-0663.80.4.514

[52] Stipek DJ Kowalski PS Learned helplessness in task-orienting versus performance-orienting testing conditions. J Educ Psychol (1989) 81(3):384 10.1037/0022-0663.81.3.384

[53] Zimmerman BJ Self-regulation involves more than metacognition: a social cognitive perspective. Educ Psychol (1995) 30(4):217–21. 10.1207/s15326985ep3004_8

[54] Newell KM Constraints on the development of coordination. Motor Dev Child Aspect Coord Control (1986) 34:341–60.

[55] Epstein JL Effective schools or effective students: dealing with diversity. In: Hawkins R MacRae B, editors. Policies for America’s Public Schools. Norwood, NJ: Ablex (1988). p. 89–126.

[56] Clark JE On the problem of motor skill development. J Phys Educ Recreat Dance (2007) 78(5):39–44. 10.1080/07303084.2007.10598023

[57] Logan SW Webster EK Getchell N Pfeiffer KA Robinson LE Relationship between fundamental motor skill competence and physical activity during childhood and adolescence: a systematic review. Kinesiol Rev (Champaign) (2015). 10.1123/kr.2013-0012

[58] Robinson LE Stodden DF Barnett LM Lopes VP Logan SW Rodrigues LP Motor competence and its effect on positive developmental trajectories of health. Sports Med (2015) 45(9):1273–84. 10.1007/s40279-015-0351-6 26201678

[59] Logan S Robinson L Wilson A Lucas W Getting the fundamentals of movement: a metaanalysis of the effectiveness of motor skill interventions in children. Child Care Health Dev (2012) 38(3):305–15. 10.1111/j.1365-2214.2011.01307.x 21880055

[60] Blair C Razza RP. Relating effortful control, executive function, and false belief understanding to emerging math and literacy ability in kindergarten. Child Dev (2007) 78(2):647–63. 10.1111/j.1467-8624.2007.01019.x 17381795

[61] Hill JL Brooks-Gunn J Waldfogel J. Sustained effects of high participation in an early intervention for low-birth-weight premature infants. Dev Psychol (2003) 39(4):730. 10.1037/0012-1649.39.4.730 12859126

[62] Reynolds AJ Temple JA Robertson DL Mann EA. Long-term effects of an early childhood intervention on educational achievement and juvenile arrest: a 15-year follow-up of low-income children in public schools. JAMA (2001) 285(18):2339–46. 10.1001/jama.285.18.2339 11343481

[63] Calkins SD Leerkes EM Early attachment processes and the development of emotional self-regulation. In: Vohs KD Baumeister RF, editors. Handbook of Self-Regulation: Research, Theory, and Applications [Internet]. New York, NY: Guilford Press (2006). p. 324–39.

